# Interhospital variations in practice and technical outcomes for endoscopic resection of early oesophagogastric adenocarcinoma: multicentre CONGRESS data set analysis

**DOI:** 10.1093/bjsopen/zrag039

**Published:** 2026-05-22

**Authors:** Kirsty Cole, Pradeep Bhandari, James A Gossage, Natalie Blencowe, Swathikan Chidambaram, Tom Crosby, Neil M Davies, Richard P T Evans, Ewen A Griffiths, Sivesh K Kamarajah, Sheraz R Markar, Nigel Trudgill, Timothy J Underwood, Philip H Pucher, the CONGRESS Collaborative, T Abdelrahman, H Haboubi, K Akbari, N Bird, E McLaughlin, J Sultan, B Alkhaffaf, K Bhatti, I Ghazzi, L Alexandre, M T Ko, B Kumar, T Ritchie, H Ali, C Tang, A Alwis, D Chan, A Athanasiou, E Best, A Elshaer, N Elzahed, A Boddy, M Bonomaully, P Healy, A Botros, C Ji, K Siggens, S Rahman, J Straatman, L Brown, I Penman, R Skipworth, M Soupashi, B Byrne, L Giet, S Lindley, T Whittaker, R Byrom, Z Evison, M White, B C Aguilera, G Nana, S L Parsons, R Vohra, C T P Choh, N Dempster, H Clements, M Owusu-Ayim, P Patil, P Coe, M Martin, L Crocker, A Cross, S Wahed, V Dayashetty, I Sarantitis, N Sharafi, A Sinha, P Turner, A Dermanis, M Di Pietro, A Jain, R O'Neill, S Pan, J Dunn, S Epton, S Zeki, S Dwerryhouse, M Forshaw, L Gall, G B Hanna, J Hoare, S Hong, K Moorthy, C Peters, N Slim, S Stevens, B Vadhwana, S Woodrow, F Ibrahim, A Madhavan, S Mastoridis, S Preston, C Johnson, S Khan, F Klevebro, H Maltzman, M Nilsson, J Lim, A Mahendran, D Mitton, V Wong, A Pervez, S Rajkumar, O Priest, N Thavanesan

**Affiliations:** Department of General Surgery, Portsmouth University Hospitals NHS Trust, Portsmouth, UK; School of Medicine, Pharmacy and Biomedical Sciences, University of Portsmouth, Portsmouth, UK; Department of Gastroenterology, Portsmouth University Hospitals NHS Trust, Portsmouth, UK; Department of Upper GI Surgery, Guy’s and St Thomas’ Hospital NHS Foundation Trust, London, UK; Centre for Surgical Research, University of Bristol, Bristol, UK; Department of Surgery and Cancer, Imperial College London, London, UK; Department of Oncology, Velindre University NHS Trust, Cardiff, UK; Division of Psychiatry, University College London, London, UK; Department of Statistical Science, University College London, London, UK; Department of Upper GI Surgery, Queen Elizabeth Hospital, University Hospitals Birmingham NHS Foundation Trust, Birmingham, UK; Department of Upper GI Surgery, Queen Elizabeth Hospital, University Hospitals Birmingham NHS Foundation Trust, Birmingham, UK; Institute of Immunology and Immunotherapy, University of Birmingham, Birmingham, UK; Department of Upper GI Surgery, Queen Elizabeth Hospital, University Hospitals Birmingham NHS Foundation Trust, Birmingham, UK; Nuffield Department of Surgical Sciences, University of Oxford, Oxford, UK; Department of Upper GI Surgery, Queen Elizabeth Hospital, University Hospitals Birmingham NHS Foundation Trust, Birmingham, UK; School of Cancer Sciences, University of Southampton, Southampton, UK; Department of General Surgery, Portsmouth University Hospitals NHS Trust, Portsmouth, UK; School of Medicine, Pharmacy and Biomedical Sciences, University of Portsmouth, Portsmouth, UK; Department of Surgery and Cancer, Imperial College London, London, UK

**Keywords:** oesophago-gastric cancer, endoscopic surgery, volume-outcome relationship

## Abstract

**Background:**

Endoscopic resection (ER) offers organ-preserving, potentially curative treatment for early (T1 N0) oesophagogastric (OG) adenocarcinoma, yet the extent of interhospital variation in practice is unclear. This study assessed variations in clinical practice and outcomes of ER for OG cancer across centres using a large multicentre data set.

**Methods:**

A retrospective analysis was conducted using the CONGRESS database, a UK-based international multicentre registry of patients with T1 N0 OG cancer between 2015 and 2022. Demographics, pathology, and outcomes for patients treated with ER were analysed. Centres were ranked according to ER volume, and patients were stratified into tertiles (low-, medium- and high-volume) with comparable numbers of patients in each group. Outcomes between low- and high-volume centres were compared using non-parametric tests and multivariable regression. The primary outcomes of interest were rates of R1 resection, procedural complications, and progression to surgery.

**Results:**

In all, 1215 patients from 28 centres were included. The median ER volume per centre for OG cancer was 72 (interquartile range 43–150) over the 8-year period. R1 resection rates ranged from 0 to 67% (mean 28.1%, median 26.5%), and complication rates ranged from 0 to 50% (mean 7.8%, median 4.0%). Patients in the high-volume tertile had lower rates of R1 resection (17.3% *versus* 26.4%; *P* = 0.001), complications (3.8% *versus* 8.2%; *P* = 0.007), and progression to surgery (9.8% *versus* 20.7%; *P* < 0.001) than patients in the low-volume tertile. These differences remained after adjustment for patient and tumour variables, with odds ratios of 0.63 (95% confidence interval (c.i.) 0.42 to 0.94; *P* = 0.023), 0.42 (95% c.i. 0.22 to 0.79; *P* = 0.007), and 0.40 (95% c.i. 0.26 to 0.63; *P* < 0.001) for R1 resection, procedural complications, and subsequent surgery, respectively.

**Conclusion:**

This study highlights significant interhospital variation in clinical outcomes for ER in OG cancer. A greater understanding of underlying factors is needed to optimize patient outcomes.

## Teaser

Using the CONGRESS multicentre registry (2015–22), this study evaluated interhospital variation in outcomes following endoscopic resection (ER) for T1 N0 oesophagogastric adenocarcinoma. Among 1215 patients from 28 centres, high-volume centres demonstrated significantly lower rates of R1 resection, procedural complications, and progression to surgery compared with low-volume centres, even after adjustment for case mix. These findings indicate substantial practice variation and suggest a volume-outcome relationship in ER for early OG cancer.

## Introduction

Endoscopic resection (ER) is recommended as the standard of care for early (T1 N0) oesophagogastric (OG) cancer^1,2^. ER allows full histopathological assessment of tumours and offers organ-preserving curative therapy in low-risk lesions. Tumours with high-risk features, such as deep submucosal invasion or lymphovascular invasion (LVI), are at increased risk of lymph node metastasis (LNM)^3^, and may be subsequently considered for radical oesophagectomy with lymphadenectomy to ensure full oncological clearance^1,4,5^. ER is a highly specialized and technically challenging procedure, where adverse events or incomplete (R1) resection may affect patient staging or treatment pathways. The nature of interhospital variation in the clinical practice and granular outcomes after ER are not well described.

The Endoscopic Resection, Esophagectomy or Gastrectomy for Early Esophagogastric Cancers (CONGRESS) international database represents the largest available data set for early OG cancer, containing demographic, tumour, and outcome variables^6,7^. The aim of the present study was to analyse interhospital variations in practice and outcomes after ER for early OG cancer.

## Methods

### Co-ordination of data collection

CONGRESS is a retrospective multicentre internally validated^8^ cohort study of patients who received treatment for early (T1 N0) OG cancer between 2015 and 2022 across centres in the UK and Sweden. Data captured include patient demographics, clinical and pathological staging, treatment details, and clinical outcomes. The complete CONGRESS inclusion criteria have been described in detail elsewhere^6,7^. For the present study, two further UK centres provided ER data in addition to the existing CONGRESS data set, using the same methodology. Only patients who underwent ER with curative intent were included in this study. Patients with squamous cell carcinoma and other tumour types were excluded, as were patients who received palliative or only surgical treatment. Inclusion was limited to patients with complete ER pathology and primary outcome data.

### Outcome measures and covariates

The primary outcome measures were R1 resection (defined as malignant cells present at the deep margin), procedural complications, and rates of subsequent surgery. Because a proportion of ER procedures were performed using a piecemeal technique, in which circumferential margin positivity is not uncommon and of lesser clinical relevance^9,10^, only deep margin positivity was assessed as an outcome in the present study. Covariates included patient factors (age, sex, and Charlson co-morbidity score), endoscopically resected pathology factors (depth of invasion, presence of LVI, and grade of cell differentiation), and procedure type (endoscopic mucosal resection (EMR) or endoscopic submucosal dissection (ESD)). Biopsy-proven cancer not visible on staging investigations (cTx, clinical Tx) was grouped with cT1 disease for analysis given the typically identical management strategies for these groups with reference to ER.

### Statistical analysis

Data were analysed using SPSS^®^ version 30 (IBM, Armonk, NY, USA). Demographic and outcome data were assessed using descriptive statistics. To assess interhospital variation, the methods previously described by Ghaferi *et al*.^11,12^ in their examination of variations in clinical outcomes after major surgery were adapted for the present study. Specifically, patients were ranked by their centre’s ER volume, and then stratified into low-, medium- and high-volume tertiles, keeping comparable patient numbers within each tertile. Because the primary objective of this study was to evaluate whether clinically meaningful differences in outcomes were present between centres at the extremes of case volume, clinical outcomes were compared between the low- and high-volume tertiles using appropriate non-parametric tests (χ^2^ and Mann–Whitney *U* tests).

Logistic regression analyses were performed for R1 resection, procedural complications, and rates of subsequent surgery, controlling for patient and tumour variables as covariates. ER technique (EMR *versus* ESD) was considered but not included in the primary logistic regression analyses because it was felt differences in the choice of technique could represent a unit practice-related confounder and therefore methodologically inappropriate to include in a statistical analysis. Instead, ER technique was included in a separate sensitivity analysis, as detailed in the *Supplementary material*. Because LVI was not formally added to guidelines as an indication for surgery until 2023 (after the study period), it was not used as a covariate in the analyses.

The statistical precision of the results of this study was interpreted using 95% confidence intervals; in addition, statistical significance was set at *P* < 0.05. This study used anonymised data from the CONGRESS collaborative^6^. CONGRESS was reviewed locally by each participating institutional authority and registered as a retrospective audit of anonymised outcome data. Approval from a research ethics committee and participant consent were therefore not required.

## Results

### Patient and hospital demographics

In all, 1215 patients were included in the present study. Patient demographics are presented in *Table 1*. The median case volume per centre over the 8-year period was 72 (interquartile range 43–150). Age, sex, and Charlson co-morbidity scores were comparable between the low- and high-volume tertiles. Although there were differences in clinical staging, with a greater number of patients with dysplasia in low- than high-volume centres (27.6 *versus* 16.6% *P* < 0.001), there was no significant difference in final pathology staging between the two groups (*P* = 0.065). Most patients (78.2%) were managed with EMR, with a greater use of ESD in high-volume centres (16 *versus* 8.4%; *P* < 0.001). Rates of ESD were significantly higher in the medium volume tertile (44.7%) than in the other two tertiles.

**Table 1:** Patient demographics, tumour staging, and procedural data across centres by ER volume tertile

	All centres	ER volume tertile			P* (low *versus* high volume tertile)
		Low	Medium	High	
No. of patients†	1215	450	365	400	
No. of centres	28	20	5	3	
Total ER volume per centre, median (i.q.r.)	72 (43–150)	38 (25–43)	72 (68–96)	150 (150–154)	< 0.001
Age at diagnosis (years), median (i.q.r.)	70 (64–77)	70 (64–77)	70 (65–77)	70 (74–77)	0.760
**Sex**					0.992
Male	950 (78.2%)	352 (78.2%)	286 (78.4%)	312 (78.0%)	
Female	265 (21.8%)	98 (21.8%)	79 (21.6%)	88 (22.0%)	
**Charlson score**					0.274
0	603 (49.6%)	212 (47.1%)	201 (55.1%)	190 (47.5%)	
1	354 (29.1%)	126 (28.0%)	101 (27.7%)	127 (31.8%)	
≥ 2	258 (21.2%)	112 (24.9%)	63 (17.3%)	83 (20.8%)	
**Site of pathology**					< 0.001
Proximal/mid-oesophagus	91 (7.5%)	21 (4.7%)	15 (4.1%)	55 (13.8%)	
Distal oesophagus/GEJ	1004 (82.6%)	391 (86.9%)	300 (82.2%)	313 (78.3%)	
Stomach	120 (9.9%)	38 (8.4%)	50 (13.7%)	32 (8.0%)	
Pathology within Barrett’s oesophagus	931 (76.6%)	344 (76.4%)	265 (72.6%)	322 (80.5%)	0.249
**Initial clinical T category**					< 0.001
Dysplasia	268 (22.1%)	124 (27.6%)	78 (21.8%)	66 (16.6%)	
T1 (a/b not reported)	505 (41.6%)	180 (40.1%)	169 (47.2%)	156 (39.3%)	
T1a	316 (26.0%)	97 (21.6%)	84 (23.5%)	135 (34.0%)	
T1b	115 (9.5%)	48 (10.7%)	27 (7.5%)	40 (10.1%)	
**ER technique**					< 0.001
EMR	950 (78.2%)	412 (91.6%)	202 (55.3%)	336 (84.0%)	
ESD	265 (21.8%)	38 (8.4%)	163 (44.7%)	64 (16.0%)	
**pT category**					0.067
Dysplasia	71 (5.8%)	30 (6.7%)	18 (4.9%)	23 (5.8%)	
T1a	779 (64.1%)	276 (61.3%)	221 (60.5%)	282 (70.5%)	
T1b (submucosal invasion depth not reported)	152 (12.5%)	65 (14.4%)	47 (12.9%)	40 (10.0%)	
T1b sm1 (≤ 500 µm)	121 (10.0%)	48 (10.7%)	46 (12.6%)	27 (6.8%)	
T1b sm2–3 (> 500 µm)	89 (7.3%)	29 (6.4%)	33 (9.0%)	27 (6.8%)	
T2 or greater	3 (0.2%)	2 (0.4%)	0 (0.0%)	1 (0.3%)	
**Tumour differentiation**					0.002
Well	276 (22.7%)	91 (20.2%)	107 (29.3%)	78 (29.5%)	
Moderate	533 (43.9%)	183 (40.7%)	161 (44.1%)	189 (43.9%)	
Poor	198 (16.3%)	75 (16.7%)	41 (11.2%)	82 (10.5%)	
Missing	208 (17.1%)	101 (22.4%)	56 (15.3%)	51 (12.8%)	
**Lymphovascular invasion**					< 0.001
Positive	162 (13.3%)	47 (10.4%)	60 (16.4%)	55 (13.8%)	
Negative	969 (79.8%)	351 (78.0%)	284 (77.8%)	334 (83.5%)	
Missing	84 (6.9%)	52 (11.6%)	21 (5.8%)	11 (2.8%)	

Values are *n* (%) unless otherwise stated. *Patients were grouped into low-, medium- and high-volume tertiles according to their centre ER volume. *P* values were calculated using X^2^ for categorical variables, and Wilcoxon rank-sum test for continous variables. †Patient numbers across tertiles are not equal due to stratification at the hospital level. ER, endoscopic resection; i.q.r., interquartile range; GEJ, gastro-oesophageal junction; EMR, endoscopic mucosal resection; ESD, endoscopic submucosal dissection.

### Clinical outcomes

**Fig. 1:**
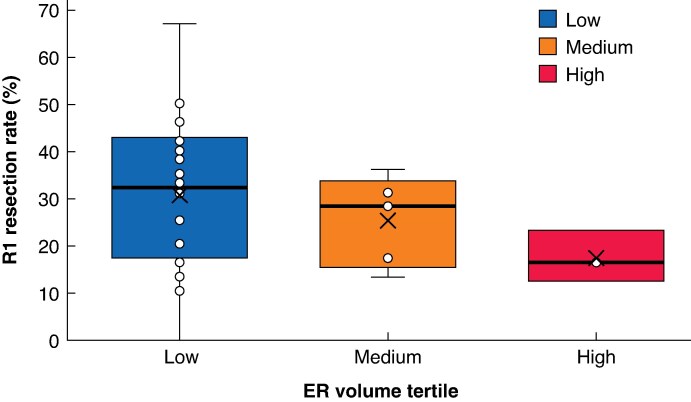
R1 resection rates across ER centres stratified into low-, medium-, and high-volume tertiles Box-plot showing R1 resection rates across low-, medium- and high-volume centres. The distribution shows higher median R1 rates and greater variability in low-volume centres, with lower rates observed in high-volume centres. Boxes show the interquartile range, with the median value indicated by the horizontal line; whiskers show the range and X symbols indicated the mean. Patients were grouped into low-, medium- and high-volume tertiles according to their centre ER volume. ER, endoscopic resection.

The overall rate of deep resection margin positivity was 22.8%. R1 resection rates varied between centres, with the high-volume tertile having lower rates than the low-volume tertile (17.3 *versus* 26.4%; *P* < 0.001; *Table 2* and *Fig. 1*).

**Table 2:** Clinical outcomes across centres by ER volume tertile (*n* = 1215)

	All centres (*n* = 1215)	ER volume tertile			P* (low *versus* high volume tertile)
		Low (*n* = 450)	Medium (*n* = 365)	High (*n* = 400)	
R1 resection	277 (22.8%)	119 (26.4%)	89 (24.4%)	69 (17.3%)	0.001
**Complication**					
Any	91 (6.7%)	37 (8.2%)	29 (7.9%)	15 (3.8%)	0.007
Perforation	10 (0.8%)	3 (0.7%)	2 (0.5%)	5 (1.3%)	
Bleeding	43 (3.5%)	17 (3.8%)	20 (5.5)	6 (1.5%)	
Other	31 (2.6%)	20 (4.4%)	6 (1.6%)	5 (1.3%)	
Progression to surgery	197 (16.2%)	93 (20.7%)	65 (17.8%)	39 (9.8%)	< 0.001

Values are *n* (%) unless otherwise stated. *Patients were grouped into low-, medium- and high-volume tertiles according their centre ER volume. *P values were calculated using X^2^ tests. ER, endoscopic resection.

**Fig. 2:**
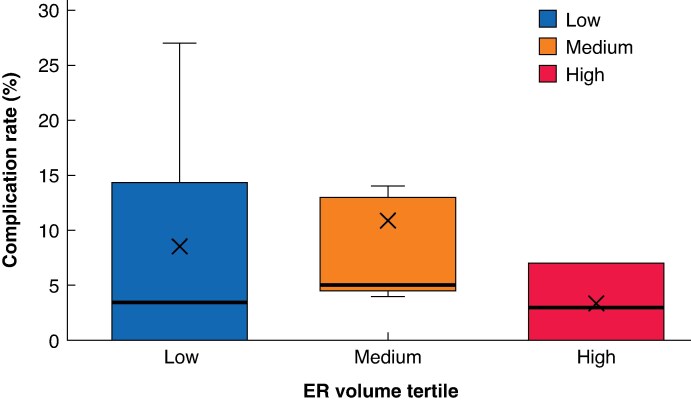
Complication rates compared across ER centres stratified into low-, medium-, and high-volume tertiles The boxes show the interquartile range, with the median value indicated by the horizontal line; whiskers show the range and ✗ symbols indicate the mean. Patients were grouped into low-, medium- and high-volume tertiles according to their centre ER volume. ER, endoscopic resection.

The overall rate of procedure-related complications was 6.7%. Complication rates were lower in the high-volume tertile than in the low-volume tertile (3.8 *versus* 8.2%; *P* = 0.007; *Table 2* and *Fig. 2*), with recorded complications including perforation, bleeding, and those related to sedation/anaesthetic.

Adjusting for patient, centre volume, and disease variables, an increased risk of R1 resection was significantly associated with more advanced T category and the low-volume tertile (odds ratio (OR) 0.63; 95% confidence interval (c.i.) 0.42 to 0.94; *P* = 0.023; *Table 3*). The risk of procedural complications was also significantly associated with centre volume tertile (high volume OR 0.42; 95% c.i. 0.22 to 0.79; *P* = 0.007; *Table 4*).

**Table 3:** Multivariable logistic regression analysis for an R1 endoscopic resection

Covariate	OR*	*P*
High-volume tertile	0.63 (0.42, 0.94)	0.023
Age	0.99 (0.97, 1.01)	0.437
**Sex**		
Male (*versus* female)	0.71 (0.45, 1.12)	0.144
**Charlson co-morbidity score**		
0	Reference	
1	1.18 (0.74, 1.87)	0.493
2	1.25 (0.76, 2.07)	0.378
**pT category**		
Dysplasia	Reference	
T1a	6.53 (0.88, 48.39)	0.066
T1b (depth not reported)	99.16 (13.07, 752.14)	< 0.001
T1b sm1	22.35 (2.88, 173.30)	0.003
T1b sm2–3	101.66 (12.93, 799.07)	< 0.001
T2 or greater	7.84 × 10^10^ (0.00, 0.00)	0.999
**Site of pathology**		
Proximal/mid-oesophagus	Reference	
Distal oesophagus/GEJ	1.76 (0.76, 4.08)	0.185
Stomach	2.68 (0.98, 7.37)	0.056

*Values in parentheses are 95% confidence intervals. OR, odds ratio; GEJ, gastro-oesophageal junction.

**Table 4:** Multivariable logistic regression analysis for the risk of complications after endoscopic resection

Covariate	OR*	*P*
High-volume tertile	0.42 (0.22, 0.79)	0.007
Age	0.99 (0.96, 1.02)	0.648
**Sex**		
Male (*versus* female)	1.26 (0.62, 2.59)	0.521
**Charlson co-morbidity score**		
0	Reference	
1	1.15 (0.59, 2.24)	0.687
2	0.90 (0.41, 1.95)	0.781
**pT category**		
Dysplasia	Reference	
T1a	0.50 (0.18, 1.40)	0.187
T1b (depth not reported)	0.86 (0.27, 2.77)	0.798
T1b sm1	1.11 (0.33, 3.72)	0.871
T1b sm2–3	0.62 (0.14, 2.79)	0.530
T2 or greater	0.00 (0.00, 0.00)	0.999
**Site of pathology**		
Proximal/mid-oesophagus	Reference	
Distal oesophagus/GEJ	0.45 (0.17, 1.18)	0.104
Stomach	1.68 (0.54, 5.22)	0.370

*Values in parentheses are 95% confidence intervals. OR, odds ratio; GEJ, gastro-oesophageal junction.

Furthermore, the rates of progression to surgery after ER were significantly lower for patients in the high- *versus* low-volume tertile (9.8 *versus* 20.7%; *P* > 0.001; *Table 2*). This remained significant after controlling for patient and tumour variables (OR 0.40; 95% c.i. 0.26 to 0.63; *P* < 0.001; *Table 5*). Increasing age (OR 0.93; 95% c.i. 0.91 to 0.96; *P* < 0.001) and a Charlson co-morbidity score ≥ 2 (OR 0.42; 95% c.i. 0.23 to 0.77; *P* = 0.005) were negatively associated with progression to surgery. Patients with deep submucosal invasion (T1b sm2–3) were more likely to progress to surgery (OR 4.03; 95% c.i. 1.42 to 11.48; *P* = 0.009).

**Table 5:** Multivariable logistic regression analysis for progression to surgery after endoscopic resection

Covariate	OR*	*P*
High-volume tertile	0.40 (0.26, 0.63)	< 0.001
Age	0.93 (0.91, 0.96)	< 0.001
**Sex**		
Male (*versus* female)	1.18 (0.71, 1.95)	0.525
**Charlson co-morbidity score**		
0	Reference	
1	0.57 (0.35, 0.93)	0.026
2	0.42 (0.23, 0.77)	0.005
**pT category**		
Dysplasia	Reference	
T1a	0.72 (0.30, 1.75)	0.471
T1b (depth not reported)	4.53 (1.75, 11.75)	0.002
T1b sm1	2.32 (0.84, 6.46)	0.106
T1b sm2–3	4.03 (1.42, 11.48)	0.009
T2 or greater	9.62 (0.67, 137.64)	0.095
**Site of pathology**		
Proximal/mid-oesophagus	Reference	
Distal oesophagus/GEJ	0.66 (0.31, 1.50)	0.282
Stomach	1.52 (0.58, 3.96)	0.396

*Values in parentheses are 95% confidence intervals. OR, odds ratio; GEJ, gastro-oesophageal junction.

Sensitivity analysis to assess the impact of choice of technique (EMR or ESD) on outcome resulted in the same significant associations, and did not demonstrate any significant relationship between technique and risk of R1 resection or complications after adjusting for centre volume and patient- and disease-specific variables (*Tables S1–S3*).

Centre characteristics by ER volume tertile are presented in *Table S4*. There were no significant differences in ER speciality (*P* = 0.521) or interventional radiology (*P* = 0.740) availability between tertiles. Mean centre bed capacity was similar across groups, although high-volume centres tended to be larger than low-volume centres (1417 *versus* 949, respectively; *P* = 0.139). The proportion of centres performing ESD increased with volume, with all high-volume centres offering ESD, although this difference did not reach statistical significance (*P* = 0.052). All centres reported that all patients undergoing ER were discussed at a multidisciplinary team meeting.

## Discussion

CONGRESS represents the largest known granular data set on ER for early OG cancer reflecting real-world practice between 2015 and 2022. The present study highlights variations in practice and outcomes, with findings suggesting that centres with higher case volumes may be associated with lower rates of procedural complications, R1 resections, and the need for subsequent surgical resection. Although previous studies have indicated a potential volume–outcome relationship for select outcomes^13,14,15^, this study is the first large-scale study to examine histological results, clinical outcomes, and subsequent treatment decisions in this context.

This study reports an association between lower ER case volume and procedural complications, a relationship that is supported by the existing literature. For example, Park *et al*.^14^ analysed a Korean database of over 90 000 patients who underwent ESD for gastric cancer. In that study, treatment in lower-volume centres was associated with significantly higher rates of ESD-related adverse events, including haemorrhage, perforation, and pneumonia (OR 0.61; 95% c.i. 0.48 to 0.77; *P* < 0.001)^14^. In another study, Markar *et al*.^13^ examined procedural volume at the level of individual endoscopists rather than institutions, finding that endoscopist workload was an independent predictor of mortality, with a clear learning curve effect wherein an increase of as few as four cases per year was associated with improved patient outcomes. These findings reinforce the importance of both institutional and operator factors in achieving optimal results with ER.

Importantly, the findings of the present study also suggest a significant association between reduced case volume and R1 resection rates, as well as an increased need for subsequent radical surgery. This is in keeping with previous studies that have examined the relationship between OG ER technical success rates and case volume^15,16^, although these studies have focused on heterogeneous populations with mixed organ ESD sites (OG, colon, and rectum) or pathologies (for example, papillomas, inlet patches), rather than dysplasia/neoplasia. Furthermore, the R1 resection rates in the present study were consistent with those reported in other endoscopic resection series^17,18^, supporting the generalizability of the present findings. Although the present data are insufficient to infer a definitive causal link between R1 rates and increased rates of surgery, this is an important possibility to consider because some referrals to surgery may have been avoided if R0 resection were otherwise achievable. However, it is also possible that higher-volume centres were less likely to appropriately recommend surgery for patients who may otherwise be offered it elsewhere. According to current guidelines, in the absence of R1 margins, the presence of deep tumour invasion or LVI may also prompt consideration for surgical resection^1^; the latter being more frequently seen in the high-volume tertile than low-volume tertile in the present study. Conversely, poor tumour cell differentiation is also considered by some to be a risk factor^4^, and this was higher in the low-volume tertile in the present study. Furthermore, the validity of current guidelines have themselves been called into question by previous studies^4,6^. This may lead to inconsistencies in clinical decision-making within local MDTs, and contribute further to the observed variation in progression to surgery rates. The present study also demonstrated significant variation in ER case volume between centres (median 72; interquartile range 43–150). It is important to note that these figures reflect only patients who met the inclusion criteria for CONGRESS (that is, T1 malignancy); actual procedural volumes, including ESDs for purely dysplastic or benign disease, are likely to be substantially higher.

There were significant differences in rates of ESD *versus* EMR between centres. Although data increasingly suggest the superiority of ESD over EMR^19,20^, practice and guidelines remain variable^1,21^ . Differences in the use of ESD for similar pT rates highlight differences in case selection and approach. The fact that differences in ESD/EMR practice are not reflected in outcome data, such as R1 rates or multivariable regression, may, in part, represent the technical challenges of ESD^22^, highlighting the need for these procedures to be performed in higher-volume centres to ensure appropriate expertise and to optimize results.

The associations noted in this study are likely multifactorial. Higher-volume centres may benefit from greater technical expertise, access to specialized resources, and streamlined workflows. In contrast, lower-volume centres often serve diverse or underserved populations and face challenges such as limited procedural demand, training opportunities, and infrastructure. Patient preferences in this setting, where intensive surveillance may be required after ER, are not clear and may affect definitive treatment plans. In addition, variations in case mix, patient demographics, and system-level factors not captured in the CONGRESS data set may introduce bias that is not accounted for. Differences in structural and process measures, such as technical approach and equipment availability, may have also affected outcomes. Although the differences in such factors (for example, the proportions of centres performing ESD) between tertiles were not significant, this is likely due to low centre numbers in each group. Other factors, such as technique and equipment, were not recorded; however, procedural volume, technical experience, and organizational set-up are often inseparably interlinked. The findings of this study also align with the well-established volume–outcome relationships evident in surgical practice, which have been a key driver for centralizing complex cancer care in the UK and other countries^23–25^. Further limitations of this study include the lack of data on individual endoscopist or overall centre ER volumes (including dysplasia), preventing any recommendations on minimum case volumes. The significant differences in the cT (preoperative) staging of disease could also infer some selection bias; for example, with triaging of suspected advanced disease to tertiary or higher-volume centres. Furthermore, improved outcomes at high-volume centres may reflect additional confounding structural factors, such as enhanced surveillance pathways for Barrett’s oesophagus and pathological reporting quality.

In conclusion, this large multicentre study highlights the variations in practice and outcomes that exist for ER of early OG cancer. These variations in outcomes emphasize the need for continued support and collaboration between centres, as well as regionalized care models to ensure equitable access to ER and the chance of organ-preserving treatment in early OG cancer. The findings of this study have potential relevance for patients, policymakers, and service providers, and may inform future strategies for optimizing ER delivery, training, and referral pathways.

## Collaborators

T. Abdelrahman, H. Haboubi (University Hospital of Wales, Cardiff, UK); K. Akbari, N. Bird, E. McLaughlin (University Hospital Coventry & Warwickshire, Coventry, UK); J. Sultan, B. Alkhaffaf, K. Bhatti, I. Ghazzi (Northern Care Alliance, Manchester, UK); L. Alexandre, M. T. Ko, B. Kumar, T. Ritchie (Norfolk and Norwich University Hospital, Norwich, UK); H. Ali, C. Tang (Mid and South Essex NHS Foundation Trust, Chelmsford, UK); A. Alwis, D. Chan (University Hospitals Plymouth, Plymouth, UK); A. Athanasiou, E. Best, A. Elshaer, N. Elzahed (Royal Sussex County Hospital, Brighton, UK); A. Boddy, M. Bonomaully, P. Healy (University Hospitals of Leicester NHS Trust, Leicester, UK); A. Botros, C. Ji, K. Siggens, S. Rahman, J. Straatman (Portsmouth University Hospitals NHS Trust, Portsmouth, UK); L. Brown, I. Penman, R. Skipworth, M. Soupashi (NHS Lothian, Edinburgh, UK); B. Byrne, L. Giet, S. Lindley, T. Whittaker (University Hospitals Bristol NHS Foundation Trust, Bristol, UK); R. Byrom, Z. Evison, M. White (University Hospitals Dorset NHS Foundation Trust, Bournemouth, UK); B. C. Aguilera, G. Nana, S. L. Parsons, R. Vohra (Nottingham University Hospitals NHS Trust, Nottingham, UK); C. T. P. Choh, N. Dempster (Oxford University Hospitals, Oxford, UK); H. Clements, M. Owusu-Ayim, P. Patil (NHS Tayside, Dundee, UK); P. Coe, M. Martin (Leeds Teaching Hospitals NHS Trust, Leeds, UK); L. Crocker, A. Cross, S. Wahed (Newcastle Hospitals NHS Foundation Trust, Newcastle, UK); V. Dayashetty, I. Sarantitis, N. Sharafi, A. Sinha, P. Turner (Lancashire Teaching Hospitals NHS Foundation Trust, Preston, UK); A. Dermanis (University Hospitals Birmingham NHS Foundation Trust, Birmingham, UK); M. Di Pietro, A. Jain, R. O'Neill, S. Pan (Cambridge University Hospitals NHS Foundation Trust, Cambridge, UK); J. Dunn, S. Epton, S. Zeki (Guy’s and St Thomas’ Hospital NHS Foundation Trust, London, UK); S. Dwerryhouse (Gloucestershire Hospitals NHS Foundation Trust, Gloucester, UK); M. Forshaw, L. Gall (NHS Greater Glasgow and Clyde, Glasgow, UK); G. B. Hanna, J. Hoare, S. Hong, K. Moorthy, C. Peters, N. Slim, S. Stevens, B. Vadhwana, S. Woodrow (Imperial College London, London, UK); F. Ibrahim, A. Madhavan, S. Mastoridis, S. Preston (Royal Surrey NHS Foundation Trust, Guildford, UK); C. Johnson, S. Khan (Sheffield Teaching Hospitals NHS Foundation Trust, Sheffield, UK); F. Klevebro, H. Maltzman, M. Nilsson (Karolinska University Hospital, Stockholm, Sweden); J. Lim, A. Mahendran, D. Mitton, V. Wong (Hull University Teaching Hospitals NHS Foundation Trust, Hull, UK); A. Pervez, S. Rajkumar, O. Priest (University Hospitals of North Midlands, Stoke-on-Trent, UK); N. Thavanesan (University Hospitals Southampton NHS Foundation Trust, Southampton, UK).

## Supplementary Material

zrag039_Supplementary_Data

## Data Availability

The data that support the findings of this study are available from the corresponding author upon reasonable request.
