# Supplementary material for: Interhospital variations in practice and technical outcomes for endoscopic resection of early oesophagogastric adenocarcinoma: multicentre CONGRESS data set analysis
Source: BJS Open. 2026 May 22;10(3):zrag039. doi: 10.1093/bjsopen/zrag039 (PMC13195632; doi:10.1093/bjsopen/zrag039)
Supplement: zrag039_Supplementary_Data [file zrag039_supplementary_data.docx]

**Interhospital variations in practice and technical outcomes for endoscopic resection of early oesophago-gastric adenocarcinoma: multi-centre CONGRESS dataset analysis**

Kirsty Cole MBBCh^1,13^, Pradeep Bhandari PhD^2^, James A Gossage MD^3^, Natalie Blencowe PhD^4^, Swathikan Chidambaram MBBS^5^, Tom Crosby PhD^6^, Neil M Davies PhD^7,8^, Richard PT Evans MBBS^9^, Ewen A Griffiths MD^9,10^, Sivesh K Kamarajah MBBS^9^, Sheraz R Markar PhD^11^, Nigel Trudgill MBBS^9^, Timothy J Underwood PhD^12^, Philip H Pucher PhD^1,5,13^

on behalf of the CONGRESS collaborative

1. Department of General Surgery, Portsmouth University Hospitals NHS Trust, Portsmouth, UK
2. Department of Gastroenterology, Portsmouth University Hospitals NHS Trust, Portsmouth, UK
3. Guy’s and St Thomas’ Hospital NHS Foundation Trust, London, UK
4. Centre for Surgical Research, University of Bristol, Bristol, UK
5. Imperial College London, London, UK
6. Velindre University NHS Trust, Cardiff, UK
7. Division of Psychiatry, University College London, London, UK
8. Department of Statistical Science, University College London, London, UK
9. Department of Upper GI Surgery, Queen Elizabeth Hospital, University Hospitals Birmingham NHS Foundation Trust, Birmingham, UK
10. Institute of Immunology and Immunotherapy, University of Birmingham, Birmingham UK.
11. Nuffield Department of Surgical Sciences, University of Oxford, UK
12. School of Cancer Sciences, University of Southampton, Southampton, UK
13. School of Medicine, Pharmacy and Biomedical Sciences, University of Portsmouth, Portsmouth, UK

**Corresponding author:**

Kirsty Cole

Department of General Surgery, Queen Alexandra Hospital, Portsmouth University Hospitals NHS Trust, Cosham, PO2 3LY, UK

Email: [Kirsty.cole13@nhs.net](mailto:Kirsty.cole13@nhs.net)

**Supplementary Materials - Index**

| **Supplementary Figures and Tables** |  |
| --- | --- |
| Table 1. Logistic regression for R1 resection rate after endoscopic resection (including EMR vs ESD as covariate) | *Page 1* |
| Table 2. Logistic regression for risk of complications after endoscopic resection (including EMR vs ESD as covariate) | *Page 1* |
| Table 3. Logistic regression for progression to surgery after endoscopic resection (including EMR vs ESD as covariate) | *Page 1* |
| Table 4. Speciality performing ER, bed capacity and IR availability by centre volume tertile | *Page 2* |

Table 1. Logistic regression for R1 resection rate after endoscopic resection (including EMR vs ESD as covariate)

|  |  |  |  |
| --- | --- | --- | --- |
|  |  |  |  |
| **Covariate** |  | **OR (95% CI)** | **p value** |
| **High volume tertile** |  | 0.61 (0.40-0.91) | 0.015 |
| **ER Technique** | ESD (compared to EMR) | 0.71 (0.39-1.27) | 0.250 |
| **Age** |  | 0.99 (0.97-1.01) | 0.476 |
| **Gender** | Male (compared to female) | 0.69 (0.44-1.10) | 0.117 |
| **Charlson comorbidity score** | 0 | ref |  |
|  | 1 | 1.20 (0.76-1.90) | 0.436 |
|  | 2 | 1.27 (0.77-2.09) | 0.357 |
| **pT** | Dysplasia | ref |  |
|  | T1a | 5.34 (0.72-39.77) | 0.102 |
|  | T1b (depth not reported) | 71.02 (9.38-537.66) | <0.001 |
|  | T1b sm1-2 | 19.57 (2.50-153.04) | 0.005 |
|  | T1b sm3 | 83.48 (10.36-672.44) | <0.001 |
|  | T2 or greater | 63.40 (6.58-611.03) | <0.001 |
| **Site of pathology** | Proximal/mid Esophagus | ref |  |
|  | Distal esophagus/GEJ | 1.82 (0.78-4.23) | 0.164 |
|  | Stomach | 2.48 (0.89-6.89) | 0.082 |
|  |  |  |  |

(Abbreviations; ER = Endoscopic resection, ESD = Endoscopic submucosal dissection, EMR = Endoscopic mucosal resection, GEJ = Gastro-esophageal junction)

Table 2. Logistic regression for risk of complications after endoscopic resection (including EMR vs ESD as covariate)

| **Covariate** |  | **OR (95% CI)** | **p value** |
| --- | --- | --- | --- |
| **High volume tertile** |  | 0.40 (0.21-0.76) | 0.005 |
| **ER Technique** | ESD (compared to EMR) | 0.65 (0.28-1.50) | 0.313 |
| **Age** |  | 0.99 (0.96-1.02) | 0.580 |
| **Gender** | Male (compared to female) | 1.33 (0.65-2.73) | 0.440 |
| **Charlson comorbidity score** | 0 | ref |  |
|  | 1 | 1.09 (0.56-2.15) | 0.797 |
|  | 2 | 0.87 (0.40-1.92) | 0.738 |
| **pT** | Dysplasia | ref |  |
|  | T1a | 0.35 (0.12-1.01) | 0.051 |
|  | T1b (depth not reported) | 0.72 (0.23-2.26) | 0.569 |
|  | T1b sm1-2 | 0.92 (0.27-3.14) | 0.891 |
|  | T1b sm3 | 0.53 (0.11-2.50) | 0.421 |
| **Site of pathology** | Proximal/mid esophagus | ref |  |
|  | Distal esophagus/GEJ | 0.44 (0.17-1.14) | 0.092 |
|  | Stomach | 1.42 (0.43-4.66) | 0.566 |

(Abbreviations; ER = Endoscopic resection, ESD = Endoscopic submucosal dissection, EMR = Endoscopic mucosal resection, GEJ = Gastro-esophageal junction)

Table 3. Logistic regression for progression to surgery after endoscopic resection (including EMR vs ESD as covariate)

| **Covariate** |  | **OR (95% CI)** | **p value** |
| --- | --- | --- | --- |
| **High volume tertile** |  | 0.41 (0.26-0.64) | <0.001 |
| **ER Technique** | ESD (compared to EMR) | 0.89 (0.45-1.75) | 0.738 |
| **Age** |  | 0.93 (0.91-0.96) | <0.001 |
| **Gender** | Male (compared to female) | 1.17 (0.71-1.95) | 0.532 |
| **Charlson comorbidity score** | 0 | Ref |  |
|  | 1 | 0.57 (0.35-0.93) | 0.025 |
|  | 2 | 0.42 (0.23-0.77) | 0.005 |
| **pT** | Dysplasia | Ref |  |
|  | T1a | 0.72 (0.30-1.75) | 0.471 |
|  | T1b (depth not reported) | 4.52 (1.74-11.71) | 0.002 |
|  | T1bsm1 | 2.34 (0.84-6.53) | 0.103 |
|  | T1bsm2-3 | 4.19 (1.44-12.20) | 0.009 |
|  | T2 or greater | 9.84 (0.68-141.94) | 0.093 |
| **Site of pathology** | Proximal/mid Esophagus | Ref |  |
|  | Distal esophagus/GEJ | 0.67 (0.31-1.41) | 0.287 |
|  | Stomach | 1.58 (0.59-4.26) | 0.365 |

(Abbreviations; ER = Endoscopic resection, ESD = Endoscopic submucosal dissection, EMR = Endoscopic mucosal resection, GEJ = Gastro-esophageal junction)

Table 4. Speciality performing ER, bed capacity and IR availability by centre volume tertile

|  | **All** | **Low Volume Tertile** | **Medium Volume Tertile** | **High Volume Tertile** | **p value** |
| --- | --- | --- | --- | --- | --- |
|  |  |  |  |  | **(low vs high)** |
| **ER speciality at centre** |  |  |  |  | 0.521 |
| Gastroenterologist | 16 (57.1%) | 11 (55.0%) | 4 (80.0%) | 1 (33.3%) |  |
| Surgeon | 4 (14.3%) | 3 (15.0%) | 1 (20.0%) | 0 (0.0%) |  |
| Both | 4 (14.3%) | 3 (15.0%) | 0 (0.0%) | 1 (33.3%) |  |
| Missing | 4 (14.3%) | 3 (15.0%) | 0 (0.0%) | 1 (33.3%) |  |
| **Centre bed capacity (mean)** | 989 | 949 | 897 | 1417 | 0.139 |
| **Performing ESD** | 14 (50.0%) | 8 (40.0%) | 3 (60.0%) | 3 (100.0%) | 0.052 |
| **IR availability** |  |  |  |  |  |
| **Everyday 24 hour** | 24 (85.7%) | 18 (90.0%) | 4 (80.0%) | 2 (66.7%) | 0.740 |
| **Weekday 24 hour** | 1 (3.6%) | 1 (5.0%) | 0 (0.0%) | (0.0%) |  |
| **Daytime only** | 1 (3.6%) | 0 (0.0%) | 1 (20.0%) | 0 (0.0%) |  |
| **Missing** | 2 (7.1%) | 1 (5.0%) | 0 (0.0%) | 1 (33.%) |  |

(Abbreviations; ER = Endoscopic resection, IR = Interventional radiology, ESD = Endoscopic submucosal dissection)
